# Relationship of menstrual irregularities to BMI and nutritional status in adolescent girls

**DOI:** 10.12669/pjms.301.3949

**Published:** 2014

**Authors:** Saira Dars, Khashia Sayed, Zara Yousufzai

**Affiliations:** 1 Saira Dars, MS, Obstetrics &Gynaecology Department, Liaquat University of Medical and Health Sciences LUMHS, Jamshoro, Sindh, Pakistan.; 2 Khashia Sayed, MBBS, SpR at St Thomas’ Hospital, London, UK.; 3 Zara Yousufzai, MBBS, MS trainee, Plastic and Reconstructive Unit, Liaquat University of Medical and Health Sciences LUMHS, Jamshoro, Sindh, Pakistan.

**Keywords:** Menstrual pattern, Body mass index, Adolescence, Anemia

## Abstract

***Objective: ***To evaluate the effect of Body Mass Index and nutritional status on the menstrual pattern in adolescent girls

***Methods:*** Four hundred one adolescent girls who attained menarche were selected from five schools in Hyderabad. The data was collected by trained medical undergraduate and postgraduates by interviewing adolescent school girls using a pre-designed pre-tested questionnaire. BMI was calculated using the formula: BMI (kg/m^2^) = Weight (kg) / Height 2 (m^2^). Hb was estimated by Sahlis method using a haemoglobinometer. Data was analyzed using SPSS 11.0.

***Results*** *:* The mean age of the girls was 14.96 +/- 1.5 years. Three hundred and five (76%) of the girls had a normal menstrual cycle, twenty-eight (7 %) had frequent periods, fifty-two (13%) had infrequent periods and sixteen (4%) of the girls had totally irregular cycles and a pattern could not be determined. Three hundred and five (76%) of girls had a normal menstrual flow, sixty-eight (17%) had heavy flow and twenty-eight (7%) had scanty flow. One hundred fifty two (38%) of girls complained of premenstrual symptoms. Two hundred thirty one (60%) girls were clinically anemic. Two hundred and seventy seven (69%) had a BMI between 18.5 - 24.9 kg/m^2.^ One hundred and eight (27%) were underweight with a BMI of 14 – 18.49kg/m^2^, while sixteen (4%) were overweight with BMI 25 – 29.99 kg/m^2^. A statistically significant relationship was found between BMI and social class (P<0.001) and BMI and menstrual pattern P<0.001).

***Conclusion*** *: *The study concludes that a majority of the girls had clinically obvious nutritional deficiency diseases. Out of the four hundred and one girls who were checked, two hundred thirty one were found to be anemic. Majority of the girls (84%) had a normal menstrual pattern, normal BMI and attained menarche before the age of 16. Overweight girls had infrequent periods.

## INTRODUCTION

Adolescence is a period of maturity, a point of physical, emotional, social and psychological change. It is considered to be the period between ages 13 and 19. Anemia is the major nutritional deficiency found in this group in Pakistan where more than 40 percent of total female population is anemic. This includes 35 percent girls of 15-19 years of age.^1^

It is rare to find a significant pathology to explain the irregularities; however it is not uncommon to find a change in pattern at that age. The pattern of menstrual cycle will have a significant impact on a girl’s reproductive life, which raises a concern for the patient and their families. It is important for clinicians as well as young patients and their parents to understand what a normal menstrual pattern is, in order to evaluate what constitutes an irregular cycle or abnormal flow. In many instances, young patients seek medical attention for menstrual irregularities, which actually fall within the normal range. 

The age of menarche is determined by general health, genetic, socio-economic and nutritional factors. The mean age of menarche is typically between 12 and 13 years.^1^^,^^2^ The initial cycles after menarche are often irregular with a particularly greater interval between first and second cycle. The early menstrual cycles are thought to be anovulatory, with frequency of ovulation being related to time since menarche and age at menarche.^3^

Most women bleed for 2 to 7 days during their first menses.^4^ Most normal cycles range from 21 to 45 days, despite variability even in the first gynecologic year, although short cycles of fewer than 20 days and long cycles of more than 45 days may occur. By the third year after menarche, 60% to 80% of menstrual cycles are 21 to 34 days long, as is typical of adults.^4^^,^^5^ BMI as classified by WHO describes having <16kg/m2 as severe underweight, 16.0 – 16.9kg/m^2 ^as moderate underweight and 17.0 – 18.49kg/m^2^ as mild underweight. Normal BMI range is 18.5 – 24.99 kg/m^2^. Anything > 25 kg/m^2^ is considered to be overweight, with 25 – 29.99 kg/m^2^being classified as pre-obese and >30 kg/m^2^ as obese.

Menstrual problems are generally perceived as only minor health concern and thus irrelevant to the public health agenda particularly for women in developing countries who may face life threatening condition. Menstrual cycle is normal physiological process that is characterized by periodic and cyclic shedding of progestetional endometrium accompanied by loss of blood which is additional vital sign adds a powerful tool to the assessment of normal development and the exclusion of pathological conditions in adolescent and young girls.^6^ Some variety of menstrual dysfunction occurs in adolescent girls which may affect normal life of adolescent and young adult women. Physical, Mental, Social, Psychological, Reproductive problems are often associated with menstrual irregularities and menstrual problems. Due to change in life style, habits, diet, the prevalence of obesity has increased in developed world which results in decreased age at menarche. ^7^

Low level of hemoglobin and nutritional status is frequently correlated with irregularities of menstrual and problems among the females in different age groups. 

The objective of this study was to evaluate the effect of Body Mass Index and nutritional status on the menstrual pattern in adolescent girls. Four hundred and one adolescent girls who attained menarche were selected from five schools in Hyderabad.

## METHODS

The current study was a cross sectional study carried out between April to June 2011. The study was conducted in Hyderabad, Pakistan where a total of four hundred and one adolescent girls aged 12 – 18 from five schools were selected after getting permission from their parents. The school’s Headmistresses were contacted via the Director of Secondary Education for permission to allow the questionnaires to be distributed among the school girls. The data was collected by trained medical undergraduate and postgraduates by interviewing adolescent schoolgirls using a pre-designed pre-tested questionnaire.

The questionnaire consisted of age, residential address, fathers occupation and income, age of menarche, date of last menstrual period, details of menstrual cycle, including cycle length, number of days the period lasts, menstrual flow (i.e. scanty, normal or heavy), presence or absence of dysmenorrhea, premenstrual symptoms such as headache, giddiness, leg cramps and abdominal cramps, and any other symptoms such as diarrhea or vaginal discharge were noted.

Clinical examination was conducted at the same time by the trained medical staff. Examination included looking for signs of anemia, lymphadenopathy, checking the thyroid gland. 

All those who were married, had primary or secondary amenorrhea, and genital tract surgery, chemo or radiotherapy or was on oral contraceptive pills (OCP) were excluded. Height and weight was also measured. BMI (body mass index was calculated using the formula: BMI (kg/m 2) = Weight (kg) / Height 2 (m^2^). Hemoglobin (Hb) was estimated by Sahlis method using a haemoglobinometer. Data was analyzed using SPSS 11.0.

## RESULTS

A total of four hundred and one girls participated in the study. The mean age of the girls was 14.96 with a standard deviation of 1.5 years (range 12 – 18 years). The socioeconomic status was determined by their father’s occupation and monthly income. 47% of the fathers worked in the public sector and 2% worked in the private sector, 39% were manual workers and 12% did not mention the status of their father’sjob.71% of the girl’s fathers belonged to the lower middle class status with a monthly income between Pakistani rupees (Rs.) 2000 – 7000. 26% belonged to the middle class with an income of Rs.7000 – 12,000 while 3% of the girls belonged to a low socio economic status with father’s income between 1000 – 2000 rupees per month.

***Age of Menarche: ***67.33 % of the girls had their first menstrual period between the age of 11 and 13 (mean = 12.92 years, SD of 1.41 years). All the girls had experienced menarche by the age of 16 years. None of the girls had primary amenorrhea.

***Menstrual Pattern: ***Three hundred and five (76%) of the girls had a normal menstrual cycle of 3-7/ 26 – 31 days. Twenty-eight (7 %) had frequent periods 5 – 8 / 22 – 28 days. Fifty-two (13%) had infrequent periods (3 -5/35 – 90) and sixteen (4%) of girls had totally irregular cycles and pattern could not be determined. After excluding these sixteen girls, with irregular patterns, the mean cycle length of the remaining three hundred and eighty five girls was 28 – 29 days. SD 12.45 days. Other details related to menstrual problems are given in Table-I.

***Nutritional Status and Anaemia: ***Nutritional status of the girls was determined by their Body mass index (BMI). The mean BMI was19.65kg/m2 SD = 2.41 kg/m2).The results are shown in the chart below . Table-II

***Anemia: ***Two hundred and thirty one (60%) girls were clinically anemic with an Hb<12g/dl. The mean Hb was 9.88g/dl, SD 2g/dl. One hundred and sixty (40%) girls had Hb 12 – 14g/dl. Of note, 8.3% girls had an Hb as low as 5.7g/dl.

***BMI and menstrual pattern: ***75.51% girls with BMI 14-24.9 had a normal menstrual pattern. All sixteen girls with a BMI of 25 – 29.9 kg/m^2^had infrequent cycles. A statically significant relationship was observed between BMI and menstrual pattern. (df = 6, x3= 116.5, P <0.001).

***Other Problems: ***Other problems identified were excessive vaginal discharge, with or without foul smell and itching 43%, abdominal pain on and off 28.18%, goiter 11%, cervical lymphadenopathy 6%, diarrhea 5% and urinary symptoms 5%.62% had primary dysmenorrhea.

## DISCUSSION

Our study concluded that there was a statistically significant relationship observed between BMI and menstrual pattern. The results showed that 75.51% of girls with BMI 14-24.9 had a normal menstrual pattern. All sixteen girls with a BMI of 25 – 29.9 kg/m^2^had infrequent cycles.

In the present study, the mean age at menarche of young girls was found to be 12.92 ± 1.41 years, which is similar to other studies.^8^^-^^12^ By 15 years of age, 98% of females will have had menarche.^13^ All the adolescent girls in the present study attained menarche before the age of 16, therefore none had primary amenorrhea. Chumlea et al^13^ and Thomas et al^14^ concluded that society's socioeconomic status can have an influence on the age of menarche as well as the prevalence of menstrual irregularities in the population. In respect of regularity of menstrual cycle, it revealed that it was regular in three hundred and five (76%) girls, whereas ninety-six (24%) had irregular cycle this is comparable to other studies.^12^^,^^15^

**Table-I:** Menstrual problems among adolecent school girls with BMI groups Relationship

*Dysmenorrhea (N = 401)*	
Yes	249 (62%)
No	152 (38%)
Cycle (N = 401)	
Regular	305 (76%)
Irregular	96 (24%)
Menstrual Days (N = 401)	
1 – 5 days	285 (71%)
> 5 days	116 (29%)
Menstrual flow (N = 401)	
Normal	305 (76%)
Heavy	68(17%)
Scanty	28 (7%)
Premenstrual symptoms (N = 401)	
Yes	153 (38%)
No	248 (62%)
Adolecent girls	BMI Index
277	1 8.5 -24.99 (69%)
16	25 – 29 (4%)
108	14-18 (27%)
BMI Status	BMI Mean±SD
69% Normal	19.65±2.41kg/m^2^
4% Overweight	
27% Underweight	

**Table-II:** Body mass index (BMI).

	*BMI (kg/m2)*
<18.49	108
18.5 - 24.99	277
25 - 29.99	16

The menstrual flow, was found to be normal in three hundred and five (76%) of the girls, while it was scanty in twenty eight (7%) and heavy in sixty eight (17%) of the girls, in contrast to a study by Begum J et al^9^ which showed a higher percentage of girls to have scanty flow and lower percentage of those with heavy flow. Dysmenorrhea is one of the commonest problems in this age group, as reported by other researchers.^16^^,^^17^ This study showed that two hundred and forty nine (62%) girls reported to have primary dysmenorrhea. This can be compared with the study findings of Begum J^8^ et al and Chowdhury et al.^18^

There have been studies, which have emphasized the importance of Body Mass Index (BMI) as an index of nutritional assessment.^19^^,^^20^ In this study nutritional status of the girls was determined by their BMI. Two hundred seventy seven (69%) had a BMI between 18.5- 24.9 kg/m2, one hundred and eight (27%) were underweight with a BMI of 14 – 18.49kg/m^2^, while sixteen (4%) were overweight with BMI 25 – 29. 9 kg/m2 (mean BMI 19.65kg/m2 SD = 2.41 kg/m2). There have been 2 large studies by Karlberg and Wang^21^^,^^22^ that have confirmed earlier onset of puberty related to a higher gain in BMI. Other studies^23^^-^^24^ reported later appearance of Menarche, menstrual cycle disorders and problems with conception, related to reduced body fat and weight loss. In our study 75.51% girls with BMI 14-24.9 kg/m^2^had a normal menstrual pattern. All sixteen girls with a BMI of 25 – 29.9 kg/m^2^had infrequent cycles (oligomenorrhea).

Anemia affects approximately 30% to 55% of adolescents of all over the world.^25^ It is particularly more pronounced in adolescents in this age group due to the physical changes that occur at puberty, utilizing a large portion macronutrients, vitamins, and minerals and tend to have an increasing need for energy, especially during the growth spurt.^26^ Two hundred and thirty one (60%) girls in our study were clinically anemic with Hb<12 g/dl. Mean Hb was 9.88g/dl with SD 2g/dl. From the two hundred and forty one(60%) girls, thirty-three of the (8.3%) girls had Hb+/- 5.7g/dl, showing significant anemia. One hundred and sixty (40%) girls had Hb 12 – 14g/dl. This shows that a large proportion of girls are anemic and the diagnosis of anemia and certain hematological disorders is often missed. The problem arises due to the lack of seeking medical attention. This in turn leads to under diagnosis of certain conditions like polycystic ovarian syndrome and endometriosis, which if untreated may have significant effects on both the reproductive and general health of women. Certain endocrinological abnormalities may be missed or delayed, making these conditions and their sequelae more difficult to treat at a later date. This may partially be due to lack of knowledge and education amongst school going girls in Hyderabad. However the reason behind the low numbers of girls seeking treatment needs to be investigated further. It is possible that the girls tend to assume that their menstrual pattern is normal and therefore do not report them at the regular school health check-ups due to lack of proper information.^27^

 Due to lack of knowledge, education, male dominance majority of adolescent girl and young women do not seek the health care services, at the same time high prevalence of malnutrition among adolescent girls results in increased reproductive problems in young women. Problems with menstrual pattern may affect 75% girls, and are the major cause of recurrent short term school absenteeism in female college students ^28^. A number of medical conditions can cause irregular or missed menses which are diagnosable and treatable even at peripheral level in early stage but this part of women’s health was neglected by primary health care. More than 90% menstrual problems are preventable which need early detection and early treatment by appropriate methods. Effectiveness of any health programme evaluated on the basis of improvement in general health of community. 

Appropriate health education measures need to be put into place to prevent this trend. Since most adolescent girls are at school going age, the initial steps to promote awareness must start in schools.

## CONCLUSION

The study concludes that a majority of the girls had clinically obvious nutritional efficiency diseases. Problems related to menstruation are quite frequent and often result in the interruption of the daily routine of the adolescent girls, therefore it is important that school officials and school health programme staff recognize these problems and need to be sensitive to their problems. Further studies should be performed to determine the reason for this trend, and newer strategies need to be employed.
